# Reducing hospital readmissions amongst people experiencing homelessness: a mixed-methods evaluation of a multi-disciplinary hospital in-reach programme

**DOI:** 10.1186/s12889-023-16048-1

**Published:** 2023-06-12

**Authors:** Stephen Malden, Lawrence Doi, Lauren Ng, Fiona Cuthill

**Affiliations:** 1 Scottish Collaboration for Public Health Research and Policy, University of Edinburgh, Edinburgh, EH8 9AG Scotland; 2 College of Medicine and Veterinary Medicine, University of Edinburgh, Edinburgh, Scotland; 3 Nursing Studies, School of Health in Social Science, University of Edinburgh, Edinburgh, Scotland

**Keywords:** Homelessness, Hospital admission, Multidisciplinary care, Secondary care

## Abstract

**Introduction:**

People experiencing homelessness are at increased risk of experiencing ill-health. They are often readmitted to hospital after discharge, usually for the same or similar reasons for initial hospitalisation. One way of addressing this issue is through hospital in-reach initiatives, which have been established to enhance the treatment and discharge pathways that patients identified as homeless receive after hospital admission. Since 2020, the Hospital In-reach programme (which involves targeted clinical interventions and structured discharge support) has been piloted in two large National Health Service (NHS) hospitals in Edinburgh, United Kingdom (UK). This study describes an evaluation of the programme.

**Methods:**

This evaluation used a mixed method, pre-post design. To assess the effect of the programme on hospital readmission rates from baseline (12 months pre-intervention) and follow-up (12 months post-intervention), aggregate data describing the proportions of homeless-affected individuals admitted to hospital during the evaluation period were analysed using Wilcoxon signed rank test, with level of significance set at p = 0.05. Qualitative interviews were conducted with fifteen programme and hospital staff (nurses, general practitioners, homeless link workers) to assess the processes of the programme.

**Results:**

A total of 768 referrals, including readmissions, were made to the In-reach programme during the study period, of which eighty–eight individuals were followed up as part of the study. In comparison to admissions in the previous 12 months, readmissions were significantly reduced at 12 months follow-up by 68.7% (P = 0.001) for those who received an in-reach intervention of any kind. Qualitative findings showed that the programme was valued by hospital staff and homeless community workers. Housing services and clinical staff attributed improvements in services to their ability to collaborate more effectively in secondary care settings. This ensured treatment regimens were completed and housing was retained during hospital admission, which facilitated earlier discharge planning.

**Conclusions:**

A multidisciplinary approach to reducing readmissions in people experiencing homelessness was effective at reducing readmissions over a 12-month period. The programme appears to have enhanced the ability for multiple agencies to work more closely and ensure the appropriate care is provided for those at risk of readmission to hospital among people affected by homelessness.

**Supplementary Information:**

The online version contains supplementary material available at 10.1186/s12889-023-16048-1.

## Background

People experiencing homelessness (PEH) are at a higher risk of cancer, cardiorespiratory disease, communicable disease, all-cause mortality and hospitalisation than the general population [1–5]. This is primarily due to a combination of factors, such as a lack of adequate shelter, pre-existing physical and mental health conditions, and higher rates of drug and alcohol use in addition to a host of systemic barriers including stigmatisation and lack of language/literacy support among others [6]. This can make both accessing appropriate healthcare and maintaining a healthy lifestyle difficult for PEH, culminating in an increased need for acute and emergency healthcare [6–8].

A significant issue that persists with PEH patients is the frequency of readmission to hospital after discharge, often for the same or similar reasons for initial hospitalisation [9–11]. Reasons for this are multifactorial, as while initial poorer health is a factor, there is evidence of other social, organisational and infrastructural factors that may lead to repeat admissions. For example, epidemiological studies have shown that both initial hospital admissions and readmission rates are significantly higher for people classed as homeless than non-homeless individuals from low socioeconomic status (SES) groups, a population that has similar incidence of poor health [10, 11]. A cohort study demonstrated that PEH had an emergency readmission rate 3.77 times higher (95% CI: 3.46–4.10) and a 12-month readmission risk of 59% compared to 20% for non-homeless low SES individuals [10].

One way of addressing the effect homelessness has on hospital readmissions in the UK context are hospital in-reach programmes [9]. Hospital In-reach initiatives have been established across multiple National Health Service (NHS) hospitals in England since 2010. These programmes utilise a multidisciplinary team of general practitioners (GP), specialist nurses, in addition to outreach workers with lived experiences, to enhance the treatment and discharge ‘pathway’ that patients identified as homeless receive after hospital admission. A randomised controlled trial of one such Hospital in-reach programme (Pathway) demonstrated significantly improved quality of life scores among intervention participants compared to control (0.12 and 0.03 increases in EQ-5D-5 L scores, respectively) [12]. Additionally, while average emergency department (A&E) re-attendance within 12 months was only marginally lower in the intervention group, the proportion of individuals sleeping on the streets after discharge was significantly lower in the Hospital in-reach arm in comparison to the control (3.8% and 14.6%, respectively; P = 0.034). A more recent audit of multiple programmes nationwide further supports the effectiveness of such programmes, reporting a 66% and 37.6% decrease in hospital admissions and A&E admissions respectively, in the 90 days post Hospital in-reach implementation [13]. Furthermore, process evaluations of these programmes employing qualitative methods have added further context to the findings from the perspectives of both clinical and service delivery staff. This identified that the culture and expertise to deal with PEH patients was expanding due to Hospital in-reach, as clearer avenues for treatment and discharge were developed with a historically difficult to treat and heterogeneous patient population [14].

While a number of homelessness service programmes exist in secondary care settings in Scotland, currently no Hospital in-reach programmes have been evaluated at scale. However, a small pilot of such a programme has been carried out in two acute hospitals in Edinburgh since 2020. This study describes an evaluation of the programme in Edinburgh, Scotland (UK).

## Methodology

This study employed an exploratory sequential approach of mixed-methods, where the qualitative data and analysis received more emphasis and informed the quantitative data and analysis. A pre- and post-test design was used to test the effect of the programme on quantitative outcomes, such as hospital readmission rates and accommodation. Qualitative methods were employed to gain insight from staff and stakeholders into the processes involved in the implementation and delivery of the programme. The qualitative data was analysed prior to the analysis of quantitative data. This evaluation was conducted externally by an evaluation team from the University of Edinburgh.

### Programme context, aims and delivery

The Hospital In-reach Project was developed in response to a recognition of the high frequency PEH are hospitalised and shortly readmitted to hospital after discharge for the same or related health issues. There are several contributing factors to this.

One factor is a general lack of infrastructure and procedural mechanisms enabling homeless services, across health and housing, to remove the barriers and facilitate the health and wellbeing of PEH after discharge from hospital.

The Cyrenians is a non-profit organisation working to combat homelessness and related health and social issues within Edinburgh and the Lothians. The charity provided funding in 2020 to deliver the Hospital In-reach programme in Edinburgh. The programme aims to reduce hospital readmissions among PEH through a multicomponent intervention delivered from time of admission through to discharge, with additional follow-up in the community to stabilise the individual’s healthcare and housing circumstances.

Specifically, the project has been delivered at two large, secondary care hospitals in Edinburgh. The programme’s aims are to provide:

- Holistic approach to care at admission, including conduct of a needs assessment in preparation for discharge.
- Continuing community support after discharge until individual is stabilised and at low risk of readmission.
- Working with clinical staff to educate them on the increased needs of PEH, and to establish stronger links with secondary care and community-based homeless services.
- Development of a specialised “Hospital In-reach team” dedicated to early intervention and focused case-management consisting of general practitioners (GPs) and nurses from a specialized homelessness GP practice. In addition to provision of link workers and care navigators (specialised homelessness keyworkers who themselves have lived experience of homelessness).
- Stronger links and establishment of clear Hospital In-reach into secure housing after discharge (through existing community partnerships).

This study aimed to evaluate the implementation and delivery processes of the Hospital In-reach programme and assess the impact of the programme in relation to readmission outcomes.

The programme was first implemented in February 2020, and the evaluation study period ran for 18 months up to September 2021. This included a 6-month PEH participant recruitment/baseline period from February-September 2020, and a subsequent 12-month follow-up period from October 2020-October 2021.

### Intervention

The Hospital In-Reach programme was a multidisciplinary, multicomponent intervention which involved both clinical and non-clinical healthcare staff based across secondary care, primary care, and community sector organisations. The funding for the programme allowed for the employment of specialist community link workers (1.6–2.6 FTE), and a full-time service manager for the duration of the project. Additionally, specialist nurses and GPs already based within the participating primary and secondary care sites contributed towards pregame delivery. Purpose-built clinical decision support algorithms were also developed to assist in the detection of patients at high risk of homelessness on admission. Upon identification, patients were assessed by the appropriate member of healthcare staff and referred to one of the following interventions based on their specific needs:

One touch: consisting of one type of support or treatment. Most often assistance with securing accommodation (or signposting to services or advice).

Light touch: receiving more than one specific intervention. For example accommodation support, usually along with emergency provisions and support referral. This often also included support to access follow-up treatment, income maximisation and linking in with existing support.

Casework: The most comprehensive of the three In-Reach interventions comprised more than one intervention and ongoing support after discharge in the community, often for a number of months.

### Impact of the COVID-19 pandemic on the programme and evaluation

The implementation and delivery of the Hospital In-reach project mainly occurred during COVID-19 and this context should be considered when interpreting the findings from this evaluation. The COVID-19 outbreak, which started in the UK in March 2020, and the series of national lockdowns that ensued, placed a renewed focus on homelessness. Aside from key workers, people were not allowed on the street, which culminated in PEH being housed in various places, including hotels. The qualitative data collection for this evaluation was collected during a period when lockdowns were being eased and the quantitative data collection periods overlapped with the national lockdowns. However, COVID-19 restrictions did have a number of direct effects on intervention delivery, as during restirctions, some ward staff worked remotely, giving admitted patients longer to self-discharge than would have been typical if more staff had been on wards in person.

### Qualitative data collection

Fifteen one-to-one semi-structured interviews were conducted between March and September 2021. Participants were purposively sampled to include a majority of staff and stakeholders who worked closely with the Hospital In-reach project team over the previous year. It involved staff from both hospitals and the community setting (n = 10) and staff employed as part of the Hospital In-reach team (n = 5).

The ten interviews with hospital staff and stakeholders included a purposeful sample from housing, health and social care community services, and the acute hospitals in Edinburgh. This comprised of clinical nursing and medical staff, housing and homelessness staff, and managers, amongst other roles. All interviewees worked in close contact with the Hospital In-reach team and PEH.

An interview topic guide informed the interviews. The primary focus for staff and stakeholder interviews were on the perceived barriers and facilitators to delivery of the hospital in-reach programme, and how the programme has impacted practice and collaborative working between partner organisations. Data was collected using video (Microsoft Teams and Zoom) or telephone interviews due to the COVID-19 restrictions in place at the time.

### Quantitative data collection

Cyrenians and NHS partner organisations were responsible for the set-up, recruitment (of PEH) and delivery of the intervention, while the external evaluation was conducted by researchers from the University of Edinburgh. The Hospital In-reach team a purpose-built electronic records management system for this project and used this to send the research team aggregate and anonymized data for the evaluation. This quantitative part of the evaluation aimed to determine the impact of the Hospital In-reach project, mainly on hospital readmission rates. Historical referral data for the 12 months prior to initial referral to the Hospital In-reach programme were used from February to September 2020 (Baseline/recruitment phase). Prospective referral data were also collected by the Hospital In-reach team at six- and twelve-months follow-up (October 2020 to March 2021 and April 2021 to September 2021 respectively) for the patient cohort that were recruited following referral to the programme during the baseline period. The Hospital In-reach team anonymised and securely shared aggregate data with the research team for analysis. However, neither the funders nor Hospital In-reach programme staff were involved in any aspects of data analysis or interpretation.

By using aggregated data rather than individual data, it was not possible to identify any individuals from the data. For example, the programme team supplied the researchers with total and mean number of readmissions at each time point, percentage of male and female readmissions at each time point, proportion of patients within each housing status category (rough sleeping, hostel accommodation, emergency accommodation, private tenancy etc.) and referrals categorised by age groups.

### Data analysis

Qualitative analysis: Thematic analysis [15] was used to analyse the qualitative data. Transcripts were read and re-read by two members of the research team and then coded to develop a coding framework. One researcher (FC) independently coded the interviews from the key hospital and community staff and stakeholders. The other researcher individually coded the transcripts from the Hospital In-reach project team (LN). Both researchers then met to refine the coding framework to be used for the remaining analysis. A consensus meeting between the researchers was held to discuss the macro codes from each interview group. Similar themes from the coding were categorised together and the final research findings were identified. All transcripts were held securely on the University of Edinburgh Datasync system, where coding was applied to all transcripts, before codes were then developed into broader themes.

Quantitative analysis: Descriptive analysis using proportions were calculated for important variables across baseline (implementation-6 months), 6–12 months post- implementation and 12–18 months post- implementation. During the first 6 months of the Hospital In-reach implementation, patients referred to specific interventions on the programme (i.e. casework, light touch, or one touch) were recruited to the study cohort and followed up over the following 12 months. Due to the non-parametric nature of observed data, the percentage change in readmission rates were assessed using the Wilcoxon signed rank test to compare patient’s historical admission rates in the 12 months prior to Hospital In-reach referral with their readmission rates at follow-up (12 months post-initial referral). Significance testing was conducted using the median, however results were presented as means and proportions to protect anonymity of patients with high numbers of admissions. Level of significance for all analyses was set at p ≤ 0.05. All analyses were conducted using SPSS statistical analysis software.

## Results

### Qualitative findings

Analysis of the 15 participants’ data resulted in the following seven themes:

1. Bridging a gap between hospital and community services.
2. Ensuring better care in hospital and treatment adherence.
3. Facilitation of safe, appropriate and timely discharges into the community.
4. Improved decision making through more informed communication between community homelessness, housing and hospital services.
5. Ongoing support in the community.
6. Factors influencing the successes of the Hospital In-reach project.
  Facilitating dialogue between services.
  Knowing and respecting role boundaries.
  Tenacity and time.
7. Cautions and challenges.

Additional supporting quotes for each theme are provided in supplementary file 1.

### Bridging a gap between hospital and community services

All of the staff and stakeholder participants spoke about the multiple ways that hospital and community services struggle to work well together to meet the needs of PEH, highlighting the urgent need for a service such as the Hospital In-reach project. A lack of communication between services was identified as a key issue. There was a strong consensus among both community and hospital staff and stakeholders that the Hospital In-reach project served as an essential bridge between acute hospital and community homelessness and housing services to reduce homelessness.

‘On hospital discharge, they do not end up on the streets with their belongings in storage any more… it is definitely making an impact on actual homelessness.’ [R3, community staff/stakeholder].

Staff and stakeholder interviewees particularly benefited from the high levels of expertise and knowledge that the Hospital In-reach team had in housing and homelessness services.

‘The best thing about them [Hospital In-reach team] is their expertise and advice on housing and homelessness. They know who to ask and the system out there and they are really helpful. It saves us hours sometimes going round in circles trying to get in touch with housing and the wrong people.’ [R10, hospital staff/stakeholder].

These accounts were in stark contrast to staff experiences prior to the implementation of the Hospital In-reach project. All interviewees gave accounts of previous experiences where the health, housing and social needs of PEH had not been adequately met, which contributed to frequent readmissions to hospital. Hospital staff and stakeholders spoke of inappropriate discharges onto the streets and other unsuitable environments.

‘I do think the bottom line is we need to ensure somebody has somewhere safe to go to when they leave hospital and often, they don’t.’ [R4, hospital staff/stakeholder].

There was a general feeling expressed of being ‘driven by the system’ [R9, Hospital staff/stakeholder]. Staff and stakeholder participants expressed how difficult it was to get appropriate and timely housing resources for discharge. These interviewees asserted that there were huge pressures on the whole health and social care system to ensure a rapid turn-over of hospital beds, which resulted in unsafe and rushed discharges.

‘There’s a lot of pressure within the NHS and especially through the covid times and I think that culture unfortunately is embedded in the NHS. No matter how much we talk about looking at a person holistically, it’s what we’re all taught to do as nurses. But actually how well we practice it is something else… so I think people are aware they should be looking at the social aspects, but in practice, I don’t think they are. They are driven by the system. We need to turn over our beds. We need to get people out.’ [R8, hospital staff/stakeholder].

The difficulty seemed often to be that the patient was seen as medically fit for hospital discharge, but their multiple social issues – including suitable housing - remained unresolved:

‘I found the patient quite tricky because the consultants - from our point of view – they said that they’re good to go [for discharge] and even though the patient has capacity, yes… they were very healthy. They didn’t need OT [occupational therapy], they didn’t qualify for that and they didn’t need physio but they had nowhere to go.’ [R1, Hospital staff/stakeholder].

In addition to these system level drivers, many of the hospital staff and stakeholders gave examples of how discharges were inappropriate due to a lack of knowledge by hospital staff of appropriate community homelessness and housing services, an inability to contact the relevant services, and a lack of understanding of the needs of PEH by hospital staff. Interestingly, clinical staff and stakeholders in the hospital setting did not discuss a lack of appropriate housing as an issue, although this was recognised by the Hospital In-reach team as a key issue.

In addition, staff and stakeholders highlighted that PEH were often stigmatised in hospital and found treatment adherence difficult:

‘Quite a lot of stigma is attached to those patients [PEH] both in hospital and even the team seeing them. So if you spoke to countless unnecessary experiences of stigma on behalf of other staff because they see them … it’s quite a skill set to manage well and you don’t get managed very well in hospitals, so it’s just horrible to conform to very rigid, very structured places. The patients have to fit in with the structure that’s imposed upon them, and that can be very difficult for [people experiencing homelessness] or drug users.’ [R9, hospital staff/stakeholder].

On the community side, homelessness and housing services spoke of how they were frequently not informed of hospital admissions, which resulted in patients losing their accommodation. Several community interviewees explained how difficult it was being outside of the hospital system and identifying which member of hospital staff to contact about discharge or ongoing care.

‘Before the Hospital In-reach team were there, it really was kind of a mess getting through to the hospitals. Everybody recognises how much pressure the wards are under but sometimes trying to get through to a ward was an impossible task or obviously also for confidentiality and GDPR [General data protection regulations] reasons, they were unable to share more than surface information.’ [R6, community staff/stakeholder].

The interviews also highlighted that it is not compulsory for clients to tell landlords that they have been admitted to hospital, so landlords are often not aware of the reasons that rooms are empty. Explaining further, interviewees revealed that bed and breakfast or boarding house accommodation for PEH may not be held for more than 24 h if someone misses the evening curfew assigned by the accommodation. As a result, they may lose their accommodation. They went on to elaborate that if landlords know that somebody is in hospital, it would be possible to arrange to hold the accommodation open for longer.

The introduction of the Hospital In-reach team was seen as critical in addressing these issues. Their services were viewed as an essential bridge in connecting hospital and community services in several important ways: (1) ensuring better care in hospital and treatment adherence; (2) facilitating safe, appropriate and timely discharges into the community,3) improving communication with staff and patients, 4) enabling longer engagement by patients with treatment and services and 5) more informed decision making between the hospital and community homelessness and housing services.

### Ensuring better care in hospital and treatment adherence

Hospital staff and stakeholders frequently remarked that the work of the Hospital In-reach team ensured better care in hospital and treatment adherence. Interviewee R14 gave a clear explanation of how they see this working in practice:

‘I think that if we can get people in and get them to stay. And then [since the Hospital In-reach project has been in place] most people have successfully managed to stay to the end of their admission, which means they’ve managed to stay abstinent from both drugs and alcohol. They’ve been prescribed opiate replacement therapy, or they’ve detoxed from alcohol. They’ve had their antibiotic treatment finished and that includes IV (intravenous) antibiotics… and we’ve had people who have had severe and musculoskeletal injuries. They have continued their physio treatment and are being discharged from the hospital but also discharged in a much better kind of physical condition.’ [R14, community staff/stakeholder].

Several interviewees commented on the relational and holistic approach of the Hospital In-reach team with PEH. Building relationships with PEH, breaking down barriers and stigma and offering additional support was widely seen by the hospital staff and stakeholders as crucial to the success of the Hospital In-reach team’s work:

‘I think for some of our client group they feel very neglected and they don’t feel they’re being listened to. So having the input from people who actually do spend the time with them and actually investing in them, it does help with ward management and people stay with treatment better.’ [R4, hospital staff/stakeholder].

Some hospital clinical staff and stakeholders acknowledged how difficult they sometimes find it to engage appropriately with PEH, especially harmful drug users. In these instances, they underscored how much the Hospital In-reach team supported them:

‘They have helped a lot with us because they can help to persuade people you know or we just need to stay. A lot of people want to come into hospital and leave, yeah, but they may need IV antibiotic therapy or something, so it’s that persuasion as well that you know we just stay here. It gives us a bit of time to work in their housing and it helps them get better.’ [R4, hospital staff/stakeholder].

### Facilitation of safe, appropriate and timely discharges into the community

There was a strong consensus by staff and stakeholders that one of the biggest impacts of the programme was that the Hospital In-reach team had enabled more discharges to be delayed until appropriate housing solutions were put in place. Staff and stakeholders asserted that this resulted in much better health outcomes for patients and reduced hospital readmissions.

‘In terms of the kind of hospital in-reach [Hospital In-reach] team, there’s been that kind of huge kind of drive to delay discharges and avoiding quick discharges and we know that nowadays to get kept in hospital, you still have to be fairly acutely unwell and medically unwell for them to keep you at all.’ [R14, community staff/stakeholder].

This stood in stark contrast to the previous experience of many hospital staff, where individuals were being sent in a taxi to [primary care setting], the statutory homelessness and housing hub. Often, accommodation was not found and people were discharged onto the streets. This was particularly problematic for people discharged on a Friday afternoon when community services were closing for the weekend.

Some of the hospital staff and stakeholders also recognised the impact of the Hospital In-reach project in empowering staff on the wards to advocate for delayed discharges too.

‘It’s highlighted to the ward staff how complicated and how difficult some peoples living situations actually are, so I think it’s kind of brought it to the forefront of a lot of staff members. You know, so they’re more understanding and they will, actually certain wards anyway, and now they will delay discharge until housing is in place or until they the Hospital In-reach project have seen them.’ [R13, hospital staff/stakeholder].

Many of the staff and stakeholder interviewees highlighted that the 10 beds in the ‘step-down’ facility, Milestone House, which isa temporary accommodation used as transit until a more permanent accommodation is found (Milestone house is a separately funded service, but one that new links were facilitated with the in-reach programme), made a significant impact to enable planned discharges, especially when someone had nowhere to go or needed some additional support before moving into appropriate housing.

### Improved decision making through more informed communication between community homelessness/housing and hospital services

For the community staff and stakeholders, the most important benefit to them of having the Hospital In-reach project was that they were informed of hospital admissions and could therefore manage the housing tenancies much better. One housing manager, who deals with PEH with the more complex of issues, asserted that:

‘The Hospital In-reach team will forward us [housing service] emails just to let us know that somebody has been admitted so it just cuts through the bureaucracy and gives us another opportunity to just step in immediately and stuff’ [R3, community staff/stakeholder].

They went on to express how difficult it is to phone an acute hospital and retrieve information on whether someone has been admitted due to bureaucratic and confidentiality issues:

‘So now to know that there is a Hospital In-reach team is there and they have that link [/in the hospital] makes it easier for us to get information or pass on information and just to ensure that everybody was involved with the client so that the client is obviously always at the core of our services.’ [R3, community staff/stakeholder].

To illustrate the difference the Hospital In-reach team had made in reducing hospital -readmissions, several staff and stakeholders gave specific case-study examples of patients who had not returned to hospital following the Hospital In-reach team interventions. Some even asserted that ‘they [Hospital In-reach team] are absolutely instrumental in reducing hospital re-admissions’ [R8, hospital staff/stakeholder].

### Ongoing support in the community

Many of the hospital staff and stakeholders talked at length about the ways that the Hospital In-reach team worked with patients to ensure that they had appropriate prescriptions on discharge, which helped improve medication adherence. Furthermore, they ensured that follow-up appointments were arranged and accompanied them to these appointments if needed.

‘It [Hospital In-reach team] definitely helps with medicine adherence for sure, and just that ongoing support and encouragement.’ [R4, hospital staff/stakeholder].

‘And if somebody doesn’t… if they fall off their script. You know they’re able to help them get back on us, and they have the Contacts and they know who to go to, so I think it’s, it’s huge even with antibiotics simple things as well. Yeah, it’s just that we remind us that somebody on the outside not in a uniform going come on take your medication is really important.’ [R8, hospital staff/stakeholder].

Many of the staff and stakeholders, in both hospital and community, commented on the time the Hospital In-reach team spent with PEH and how this contributed to a much better uptake of follow-up appointments for specific patients.

‘I think a lot of my patient group would just never attend [outpatient appointments] previously so I think they [Hospital In-reach team] did manage to go to get them to continue with physio things like that and it’s all the things that we would never have happened before.’ [R13, hospital staff/stakeholder].

### Factors influencing the successes of the hospital in-reach project

The majority of the staff and stakeholders identified the relational aspects of the work of the Hospital In-reach team as central to the success of the project. These factors were namely a constant dialogue between services, knowing their role and not ‘stepping on toes’, and their tenacity and time.

### Facilitating dialogue between services

Interviewees spoke of the ways that the Hospital In-reach team adopted an inclusive approach to relationships with staff and patients, and facilitated excellent communication between and within service areas. For example, one interviewee highlighted that these improved communication channels enabled community organisations to better support PEH with their health and recovery, along with treatment adherence:

‘The consistency, the great communication, kind of from the Hospital In-reach team with us. There’s a constant dialogue with the Hospital In-reach team and the Housing First, because our paths crossed so often, so that’s certainly one of the main drives for success and there’s a real safety net.’ [R6, community staff/stakeholder].

They continued by explaining that the Hospital In-reach team made sure that everyone was involved and ‘in the loop’, including patients:

‘They [Hospital In-reach team] were in conversations and chats with the patient all the time, so it was very much like and she would keep us up to date…so there was always very clear lines of communication so you know I found it. I found it helpful.’ [R1, Charge Nurse].

One attribute that was commonly cited by the staff and stakeholder participants was the friendly, non-judgemental and approachable attitude of the Hospital In-reach team.

‘They’ve got great personalities and they’ve… they’ve definitely got the right attitude to deal with the client group.’ [R4, hospital staff/stakeholder].

### Knowing, and respecting, role boundaries

Several hospital staff and stakeholders commented on the positive ways the Hospital In-reach team worked well together and respected the role boundaries of other staff.

‘I think it is having a team where their expertise is not overlapping with my expertise and it’s entirely additional and separate and adds as much as the medical expertise to their patients outcomes, if not probably more.’ [R9, hospital staff/stakeholder].

In addition, many of the staff and stakeholders commented on the strong existing networks that the Hospital In-reach team had with housing, homelessness and community services, and the value that this brought to partnership working.

‘It’s not just knowing people in the wider third sector but also within the City Council and social work…it is the social network of people working within the wider sector.’ [R5, hospital staff/stakeholder].

### Tenacity and time

A number of staff and stakeholders drew attention to the tenacity of the Hospital In-reach team and their ability to persevere on sorting an issue out for a patient.

‘They’re very clear with people when they’re doing it, and that’s what they’re going to do. And then they follow it through.’ [R15, community staff/stakeholder].

There was also recognition that it took a considerable amount of extra time and energy to keep going and to ‘follow through’ on problems. The Hospital In-reach team were often compared to the work of social workers. Staff and stakeholders perceived that the Hospital In-reach team had more time to sort these issues.

‘If a social worker’s involved, they can’t spend that intensive time that the hospital in reach can [Hospital In-reach team]. It’s all about that discharge and that transition to somewhere else. So they are able to spend a huge amount of time in putting the things in place and I haven’t seen any other service doing that as effectively as they have, so I would like to think it would continue.’ [R14, community staff/stakeholder].

Many interviewees commented on the ‘can do’ attitude of the Hospital In-reach team:

‘The in-reach [Hospital In-reach team] are just so good at saying, OK, I’ll take that on. I’ll go and speak to the consultant or algorithm. Pick up the medications and you know that they’re very, very good at addressing as much as they can before people get even through the doors.’ [R14, community staff/stakeholder].

Nonetheless, they did caution that this approach to service delivery can be labour intensive for the team. This was identified as one of the challenges for the Hospital In-reach project.

## Cautions and challenges

Several hospital staff and stakeholders highlighted the bureaucratic difficulties that a third sector team working within an acute hospital environment encounter and the protracted length of time it takes to overcome these barriers. After over a year, the Hospital In-reach team still did not have access to the NHS medical record system. This was widely recognised by staff and stakeholders as frustrating and limiting to the work of the project. Many hospital staff also commented on how difficult it must be for the Hospital In-reach team not to have an office or base in the hospital. There were concerns that the Hospital In-reach team had to make phone calls in the middle of the corridor or in other people’s offices, which raised issues in relation to confidentiality.

‘Bureaucracy and coronavirus have been the big two barriers [for the Hospital In-reach team to overcome.’ [R5, hospital staff/stakeholder].

Following on from these concerns, some anxieties were expressed in relation to the governance structures within the project. Most staff and stakeholders knew who to contact in the Cyrenians Scotland if they had concerns with the Hospital In-reach team. However, questions were raised as to whether the Hospital In-reach team were held to the same stringent health, safety and confidentiality regulations as NHS staff.

There were ambiguities expressed by several hospital staff and stakeholders as to the exact role, responsibility and referral criteria for the Hospital In-reach team. A number of staff and stakeholders both within and out with the hospital environment acknowledged that they did not fully understand the distinction between the Hospital In-reach team and other services, particularly social work and occupational health services. There were a small number of instances where staff and stakeholders were unsure which service was the most appropriate to contact or there had been a misunderstanding between these services and the Hospital In-reach project. All interviewees who highlighted this issue asserted that this would improve as the role of the Hospital In-reach project became clearer and more established over time.

‘Maybe social work are kind of passing things on to the Hospital In-reach project, which it would maybe be more appropriate for the social work and team to be involved in.’ [R13, hospital staff/stakeholder].

Many participants who were hospital staff felt the team needed to publicise the service more. Some staff were unsure and wary at first, such as social workers and occupational therapists.

The Hospital In-reach project seemed to be well known in certain areas, such as the respiratory, orthopoedics, infectious diseases, drug and alcohol services [R13, hospital staff/stakeholder]. It was widely acknowledged that it had been difficult for the team to get to know all areas in the hospitals due to COVID-19. There was optimism that this would improve as covid restrictions were relaxed.

Several staff working within the hospital environment were concerned for the health and well-being of the Hospital In-reach team, particularly entering into a very busy acute hospital environment. One interviewee described it as a ‘baptism of fire’,

‘I don’t know whether it was a baptism of fire coming into that environment. Maybe more about how they are looked after as a team and what their needs might be in terms of, you know, training, rank, trauma, exposure, things, because even just walking into a hospital environment is quite shocking and daunting.’ [R4, hospital staff/stakeholder].

It was generally agreed among interviewees that the demand for the project outweighed supply and that the fragility of the funding would be a difficulty going forwards.

### Quantitative findings

**Table 1:** Total referrals to the Hospital In-reach programme (including multiple referrals)

Variable	Baseline (0–6 months post-implementation) n = 245 (%)	6–12 months post-implementation, n = 209 (%)	12–18 months post-implementation, n = 314 (%)
Sex			
Male	193 (79)	156 (75)	208 (66)
Female	52 (21)	51 (24)	106 (34)
Unknown	0	2 (1)	0
Age			
16–24	10 (4)	10 (5)	31 (10)
25–34	49 (20)	41 (20)	61 (19)
35–44	74 (30)	74 (35)	105 (34)
45–54	49 (20)	50 (24)	72 (23)
55–64	23 (9)	15 (7)	29 (9)
≥ 65	9 (4)	7 (3)	10 (3)
Unknown	31 (13)	12 (6)	6 (2)
Accommodation type on admission			
Tenancy	12 (5)	21 (10)	37 (12)
Temp. tenancy	53 (22)	37 (18)	65 (21)
Shared housing and (B&B)	56 (23)	50 (24)	97 (31)
Hostel/supported	29 (11)	33 (15)	33 (10)
No fixed abode	95 (39)	68 (33)	82 (26)
Intervention type			
Casework	38 (16)	16 (7)	16 (5)
Light touch	33 (13)	35 (17)	76 (24)
One touch	35 (14)	65 (31)	93 (30)
D/C prior to contact	79 (32)	39 (19)	48 (15)
Service not required	60 (25)	54 (26)	81 (26)
Total number of referrals receiving intervention (casework, light touch or one touch)	106 (43)	116 (56)	185 (60)

Table 1 shows the total number of referrals to the Hospital In-reach project, from February 2020 to October 2021. Overall, more men than women were referred to the Hospital In-reach project. However, it appears that the proportion of women referred increased over time.

Once individuals were referred to the Hospital In-reach project, support after discharge was often provided. However, across the three data points, it was clear that about a third of those referred did not require any further intervention. Additionally, the majority of those who were discharged (or self-discharged) prior to receiving any contact from the programme team were not followed up in the community due to consent issues, and inaccurate addresses in patient records. Patients who were identified as high risk by clinical decision support were followed up through links with local harm reduction teams. The majority of those discharged prior to contact (n = approx. 80%) were in an acute medical ward. At 0–6 months post implementation 32% of the referrals were discharged before being seen. This reduced to 15% at 12-18months post implementation. It appeared the proportion of those who received light touch and one touch interventions increased during that period. For instance, only 14% of the total referrals at baseline received one touch intervention, but this rose to 30% twelve-eighteen months post implementation.

**Table 2:** Mean number of hospital admissions (of any kind) in the 12 months prior to Hospital In-reach referral, 6–12 month post-implementation and 12–18 months post implementation

Intervention (number of patients)	Baseline (Mean no of Admissions 12 months prior to Hospital In-reach programme referral)	Mean no of readmissions from baseline to 6 months	Mean no of readmissions from 6 months to 12 months	Mean no of readmissions from baseline to 12 months	% change from baseline to 12 months
**All interventions (n = 66)**	3.2	0.5	0.6	1	-68.7%**
**Casework (n = 18)**	3.2	0.5	0.6	1.2	-62.5%*
**Light touch (n = 22)**	4.4	0.7	0.6	1.6	-63.6%**
**One touch (n = 26)**	2.2	0.3	0.1	0.4	-81.8%**

* = P < 0.05; ** = P < 0.01

Eighty-eight participants were referred to one of the three Hospital In-reach interventions over in the initial 6 month baseline/recruitment period and followed up. However, 17 patients had no historical admissions data available and were excluded from the analysis. A further one patient died prior to 6 month follow-up, and two died between 6 and 12 month follow up and were excluded from the interim and final analysis, respectively. A total of 66 participants were included in the overall analysis (Table 2). For the whole sample with complete admissions data, receiving any Hospital In-reach intervention resulted in a significant reduction of 68.7% in readmissions compared to the 12 months prior to initial Hospital In-reach referral (p < 0.01). For the 18 patients who received a casework intervention, a statistically significant reduction in readmissions of over 60% was observed (p < 0.05). Similarly, significant reductions in readmissions were observed in participants who received either the light touch or one touch interventions, with 63.6% and 81.8% less readmissions, respectively (both p < 0.01). Of the seventeen patients with missing historical admissions data, only one readmission was observed over the 12-month follow-up period, meaning their exclusion from the analysis unlikely have changed the results obtained.

**Table 3:** Casework interventions provided and outcomes. N = total number of referrals requiring intervention. (%) = percentage of those requiring intervention for which a successful outcome was achieved

Area of support provided	1st data collection point N = 31 (%)	2nd data collection point N = 21 (%)	3rd data collection point N = 18 (%)	Mean percentage across three time points
Completion of inpatient treatment (recommended treatment regimes completed before discharge)	27 (87)	21 (100)	13 (72)	86%
Support follow-up treatment acute (targeted support in community to complete treatment)	13 (42)	13 (62)	11 (61)	55%
Access primary care services (e.g. GP registration and attendance support)	19 (61)	19 (91)	13 (72)	75%
Appropriate accommodation sourced for discharge	24 (77)	10 (48)	4 (22)	49%
No fixed accommodation to appropriate housing sourced	15 (48)	10 (48)	13 (72)	56%

Table 3 describes the proportion of patients who were successfully managed with regards to treatment completion/support, linking with primary care, and sourcing of appropriate accommodation prior to discharge. Across the Hospital In-reach programme cohort, 86% of those receiving interventions completed their inpatient treatment course, with 55% successfully following up acute treatment, while linking patients with primary care access post-discharge was achieved in 75% of admissions. Sourcing of appropriate housing post-discharge was achieved for 49% of individuals, while 56% of patients who had no fixed accommodation at admission were discharged to appropriate housing.

## Discussion

This evaluation examined the implementation and delivery of the Hospital In-reach project from the perspective of stakeholders, such as hospital and community staff and Hospital In-reach team members. It also examined the impact of the programme in relation to readmission outcomes. During the 12 months intervention period, hospital readmissions reduced by approximately 69% compared to the 12 months prior to Hospital In-reach referral. The qualitative element of the evaluation suggested that this significant reduction in readmissions may have been due, in some part, to the work of the Hospital In-reach team acting as an essential bridge and connecting hospital secondary care services with community homelessness and housing services. The Hospital In-reach project fills a gap in service provision by enabling improved decision making through more informed communication between community homelessness and housing and hospital services. This was widely seen by staff as key in preventing discharges to inappropriate accommodation, including discharge to the streets, and reducing readmissions to hospital.

Hospital secondary care staff viewed the Hospital In-reach team as an excellent resource in enabling PEH to engage with treatment in hospital and to facilitate more planned, timely and appropriate discharges into the community. Community homelessness and housing services attributed improvements in services to their ability to readily contact the Hospital In-reach team within secondary care settings. This enabled them to be informed about admissions to hospital. It also allowed them to retain housing for PEH during this period and facilitated early planning for discharge. Similar housing initiatives have been shown to reduce hospital readmission in the literature. The apparent gap in hospital readmission rates between housed low socioeconomic status groups and homeless groups [10] indicates that the health issues faced by these groups may be exacerbated by a lack of adequate housing and support following discharge. ‘Housing first’ initiatives aim to improve access to adequate housing for high-risk PEH. A recent systematic review of randomised controlled trials of housing first initiatives found that while the results were inconclusive with regards to improved health and substance use outcomes, intervention groups were significantly less likely to use emergency departments, be hospitalised, and spent significantly less time in hospital than control groups across the four trials included in the review [14].

The results of the housing first trials appear to highlight the need for greater organisational infrastructure or structural interventions to ensure adequate housing and support is available to PEH upon hospital discharge. Additionally, there appears to be a need to ensure that the care during hospital stay is tailored to the specific needs of PEH, which may vary between individuals [8–10, 16].

The present programme was widely welcomed by all of the staff interviewed, both in hospital and community settings. The person-centred, holistic and relational approach employed by the Hospital In-reach team was viewed as critical in engaging with, and reducing admissions, for PEH. Across the period of the Hospital In-reach project, an increasing number of PEH had received either one touch or light touch interventions as they transitioned from hospital. However, during that same period, the number of PEH receiving casework interventions reduced, likely indicating that the Hospital In-reach project is contributing to reducing the number of PEH who may require more intensive intervention upon hospital discharge.

Despite the encouraging findings observed in this evaluation, there are a number of limitations to the study that should be acknowledged when interpreting the findings. Firstly, the quasi-experimental design used may have introduced bias associated with non-randomised studies. Secondly, we were unable to collect individual-level demographic data due to ethical restrictions, which limits the scope of the analysis we have conducted and our ability to demonstrate differential effects of the programme by socio-demographic factors. Specifically, we were reliant on programme staff to supply the evaluation team with this aggregated data, which could in turn increase the risk of measurement bias or reporting bias impacting the readmission rate findings. It is also possible that observed reductions in readmissions could be inflated by the inclusion of one-off admissions, where the individual is unlikely to be readmitted. Additionally, the impact that COVID-19 policies may have introduced to the typical housing patterns of PEH should not be ignored. However, by using triangulation of both qualitative and quantitative data, we have demonstrated that the results observed with regards to readmission rates may be attributed to specific components of the hospital In-reach programme, which highlights the mixed methods design of this evaluation as a strength of the study. Despite the limitations highlighted, the direction of intervention effect does indicate that the progamme reduced readmissions, which merits further exploration through a fully-powered trail, using individual-level patient data which can be appropriately analysed in order to reduce the impact of bias.

## Conclusions

This evaluation has demonstrated that the Hospital In-reach project has bridged an important gap between hospital and community services and facilitated safe, appropriate and timely discharges of PEH into the community. All stakeholders involved found the Hospital In-reach project valuable, but they also recognised the unique challenges of navigating the various bureaucratic processes in hospitals, especially for a third sector organisation. The Hospital In-reach programme also appears to be labour intensive and an economic evaluation of the programme may be warranted.

## Electronic supplementary material

Below is the link to the electronic supplementary material.

Supplementary Material 1

## Data Availability

Data pertaining to this study are available from the corresponding author upon reasonable request.

## Abbreviations

NHS: National Health Service
UK: United Kingdom
PEH: people experiencing homelessness
A &E: Accident & Emergency
GP: General Practitioner
GDPR: General Data Protection Regulation
IV: Intravenous
