# Supplementary material for: Reducing hospital readmissions amongst people experiencing homelessness: a mixed-methods evaluation of a multi-disciplinary hospital in-reach programme
Source: BMC Public Health. 2023 Jun 12;23:1117. doi: 10.1186/s12889-023-16048-1 (PMC10258765; doi:10.1186/s12889-023-16048-1)
Supplement: Supplementary file 1 — Supplementary Material 1 [file 12889_2023_16048_MOESM1_ESM.pdf]

Supplementary file 1.

Table 1. Additional supporting quotes for qualitative data themes

**Bridging a gap between hospital and community services**
'They [Hospital In-reach team] are bringing assistance to navigate the often complex bureaucracy of the health and social care system. So there's a clear benefit to the people experiencing homelessness.' [R5, hospital staff/stakeholder].
' I think it's gonna need to stay in the long term if we can help people to kind of get rehoused and stabilised and their health and their medication and they want to make positive changes. It makes sense to do that, 'cause otherwise there is the cost to in-hospital beds think…I would love it to continue as I think it has been really successful.' [R15, community staff/stakeholder].
' I hope it [Hospital In-reach programme] can continue because it's certainly an asset when they are involved. People do move on too much safer conditions and safer environment and actually have somebody shouting and arguing their case when they [PEH] are unable to, really helps too.' [community staff/stakeholder].
'I couldn't be more happier like, the [Hospital In-reach project] staff were phenomenal… absolutely phenomenal. When told about my discharge, they were there every day, or when they could, making sure that I had some accommodation from when I came out of the hospital' [LE1].
'It was always really difficult to get in touch with housing. And even if people were asking you to help them with. Like ongoing concerns or even layers of concerns. I've rolled with an issue and just going back and forward and you could never get to speak to any of the housing officers that were involved.' [R10, hospital staff/stakeholder].
'There are so many of these community services that are for patients that they [PEH] don't predominantly fit into that they need rehab..but they're younger than 65, so it's they're kind of like the forgotten group of people…so I sometimes feel like I spend my days chasing these complex discharges. oh, I wish, I wish..I wish that someone would point me in the right direction so I could at least speak to this right person.' [R1, Hospital staff/stakeholder].
**Ensuring better care in hospital and treatment adherence**
'There's also a clear benefit [of the Hospital In-reach team] to the nurses and other healthcare staff who are trying to do their best for that individual, but you lack the experience in these more social aspects around welfare benefits, housing and so on, and they don't have the time or the expertise to do that so they're almost universally delighted when the Cyrenians approach them to say hello…I think, by and large, they've been you know, warmly welcomed particularly there are particular wards and departments that see a disproportionate number of people experiencing homelessness.' [R5, hospital staff/stakeholder]
**Facilitation of safe, appropriate and timely discharges into the community**
'The outcome is just so much better [due to the Hospital In-reach project], so I suppose it gets a bit more continuity to discharges and then they're aware that someone would be discharged

within a certain time and they are able to plan and the best place for people to go.' [R10, hospital staff/stakeholder].
'A lot of patients have managed to go from hospital and continue their recuperation at Milestone. So that's been a huge help as well.' [R13, hospital staff/stakeholder].
'Since the in-reach [Hospital In-reach] team started, there's a high proportion of the people that have been in here [Milestone House] and have all gone on to stay in safe, secure accommodation… in fact, nobody's been asked to leave and sent out into nothing, so everybody's been discharged to safer accommodation,' [R14, community staff/stakeholder].
**Improved decision making through more informed communication between community homelessness/housing and hospital services**
'If they [PEH] don't return to their accommodation, so the fact that we can phone them [Hospital In-reach team] and see if somebody has been admitted makes a huge difference because they will usually tell us that so and so has been admitted, is expected to maybe be discharged and then there will be some leeway with the B+Bs' [R6, community staff/stakeholder].
'One of the things that we see it certainly that makes our service work better is that people are seen in the hospital before they come into us [Milestone House], so the engagement starts quite early on. We'll know what to expect when they're coming in, because it's already been talked about. It's already been agreed and I think that the in-reach [Hospital In-reach] team are very good at looking at what needs to be put in place to make that that transition from hospital to here much smoother and nine times out of ten, that's around their medications and prescriptions. If they're not right, people won't stay and they won't engage and so that the in-reach team are fantastic at that.' [R14, community staff/stakeholder].
**Ongoing support in the community**
'the Cyrenians have been really supportive and get as much information. I know even people. I can ask them questions anytime I want and they are you really supportive and give me the best kind of advice as well when I need to as well for somebody else that they're not working with.' [R15, community staff/stakeholder].
'being able to go to Milestone and treatment can continue at milestone so people have finished an antibiotic therapy in wound care, and hep C treatment you know and stabilized on methadone… and other pain relief.' [R13, hospital staff/stakeholder].
'The Hospital In-reach Project will go above and beyond. You know, ensuring that people attend medical appointments. Kind of the following patients and take them to kind of follow up appointments.' [R13, hospital staff/stakeholder].
Facilitating dialogue between services
'It [Hospital In-reach team]… make sure that they know everybody is in the loop.' [R6, community staff/stakeholder].

'At the end of the day as well and not just about qualifications and actually being able to engage with somebody [experiencing homelessness and/or harmful drug use] is probably the most important thing.' [R8, hospital staff/stakeholder]
Tenacity and time
'Kind of, I think they all like, dogs with bones, but for all the right reasons that cause the case for the client.' [R3, community staff/stakeholder].
'They [Hospital In-reach team] did everything – they arranged the discharge to Milestone house…they basically spoon fed me what to do.' [R1, hospital staff/stakeholder].
**Cautions and challenges**
'What you're seeing and what you understand and what you can take from what you're reading on track because it is very medicalised… I think a lot of if they're going to be doing that, I think there is a lot of support that's needed in confidentiality and information sharing, which I think we all need to be better at.' [R8, hospital staff/stakeholder].
'The demand is more than they can meet, I think so more staff yeah better more awareness around the hospital… I think this this kind of collaboration with third sector needs to grow…it is not mainstreamed at all and so that also makes it fragile going forward.' [R9, hospital staff/stakeholder].
